# The Role of Kynurenine and 5-Hydroxytryptophan in Modulating Microbiota and Their Implications in Exudative Age-Related Macular Degeneration

**DOI:** 10.3390/diagnostics16101475

**Published:** 2026-05-13

**Authors:** Alvita Vilkeviciute-Petraite, Akvile Bruzaite, Dzastina Cebatoriene, Dalia Zaliuniene, Rokas Lukosevicius, Jurgita Skieceviciene, Juozas Kupcinskas, Rasa Liutkeviciene

**Affiliations:** 1 Laboratory of Ophthalmology, Neuroscience Institute, Medical Academy, Lithuanian University of Health Sciences, 50161 Kaunas, Lithuania; alvita.vilkeviciute@lsmu.lt (A.V.-P.); rasa.liutkeviciene@lsmu.lt (R.L.); 2 Medical Academy, Lithuanian University of Health Sciences, 44307 Kaunas, Lithuania; dzastina.cebatoriene@lsmu.lt; 3 Department of Ophthalmology, Medical Academy, Lithuanian University of Health Sciences, 50161 Kaunas, Lithuania; 4 Laboratory of Clinical and Molecular Gastroenterology, Institute for Digestive Research, Lithuanian University of Health Sciences, 44307 Kaunas, Lithuaniajuozas.kupcinskas@lsmu.lt (J.K.); 5 Department of Gastroenterology, Lithuanian University of Health Sciences, 50161 Kaunas, Lithuania

**Keywords:** exudative age-related macular degeneration, metabolites, kynurenine, 5-hydroxytryptophan, microbiota

## Abstract

**Background/Objectives:** This study explores the roles of kynurenine and 5-hydroxytryptophan (5-HTP) in modulating gut microbiota and their potential implications for exudative age-related macular degeneration (AMD). By examining the interplay between these metabolites and the microbiome, we aim to uncover novel pathways that may influence the pathogenesis of AMD. Understanding these associations could lead to innovative therapeutic approaches for managing this leading cause of vision loss in the elderly. To investigate the roles of kynurenine and 5-HTP, alongside the composition of the nasopharyngeal microbiota, in patients with exudative AMD. **Methods:** Blood metabolite profiling was performed using LC–MS–based metabolomics. Metabolites were extracted with cold methanol/water containing internal standards, filtered through a 10 kDa cutoff filter, separated on a ZIC-HILIC HPLC column, and detected using an Orbitrap mass spectrometer. Metabolites were identified using MZmine 2 software. **Results:** Patients with exudative AMD exhibited a profound systemic shift in tryptophan metabolism, characterized by significantly lower plasma levels of 5-HTP and higher levels of kynurenine compared to control subjects (*p* < 0.001 for both). Logistic regression analysis confirmed that both metabolites were independent predictors of AMD status; higher kynurenine levels were associated with increased disease probability, while higher 5-HTP levels demonstrated a protective effect. The kynurenine/5-HTP ratio emerged as a robust biomarker, achieving an area under the curve (AUC) of 0.85 with an optimal threshold of 3.43 (74.1% sensitivity, 84.4% specificity). When integrated with age and gender, the diagnostic performance of the model reached an excellent AUC of 0.92. After adjusting for demographic factors, the kynurenine/5-HTP ratio remained a potent independent risk factor, with each unit increase associated with a 6.30-fold increase in the odds of exudative AMD. **Conclusions:** Exudative AMD is characterized by a shift in tryptophan metabolism toward the kynurenine pathway, with decreased 5-HTP, increased kynurenine, and an elevated kynurenine/5-HTP ratio. This ratio showed a strong independent association with AMD and excellent diagnostic performance, highlighting its potential as a biomarker and its role in disease pathogenesis.

## 1. Introduction

Age-related macular degeneration (AMD) is a major cause of irreversible central vision loss, primarily affecting individuals over 60 [1]. This condition can affect both eyes, though the severity may differ between eyes [2]. In developed countries, AMD is the leading cause of visual impairment, with over 30% of those aged 75 and older affected [2]. Statistics indicate that the prevalence of AMD rises from 0.2% in those aged 55–64 to 13% in individuals aged 85 and above [3]. With the elderly population projected to increase significantly, the incidence of AMD will likely rise, leading to greater visual impairment. AMD progresses through four distinct phases. Initially, normal aging is characterized by small drusen (under 63 μm) and an absence of pigment changes. The early AMD stage is defined by intermediate drusen (between 63 μm and 124 μm), though the retinal pigment epithelium (RPE) remains unaffected. Intermediate AMD involves widespread drusen, including at least one large deposit (125 μm or greater), often accompanied by RPE abnormalities. Finally, advanced AMD is characterized by significant vision loss due to geographic atrophy (GA) of the fovea or neovascular complications. Research by Liew has further validated the accuracy of the AREDS simplified severity scale for tracking these stages [4].

Despite decades of research, the exact pathogenesis of AMD development remains elusive, characterized by a complex interplay of modified, non-modified, and as-yet-undiscovered risk factors. While the clinical staging of the disease provides a roadmap for vision loss, recent breakthroughs have identified a systemic link between the upper respiratory tract and ocular health.

The pathogenetic association involves a nasopharyngeal-retina axis, where local microbiota dysbiosis—marked by increased abundance of genera like *Gemella* and *Streptococcus*—triggers a systemic shift in tryptophan metabolism. This shift favours the production of pro-inflammatory kynurenine metabolites, such as 3-hydroxykynurenine, over the neuroprotective 5-HTP/serotonin, ultimately promoting chronic inflammation, oxidative stress, and pathological angiogenesis characteristic of exudative AMD [5].

Furthermore, the nasopharyngeal microbiota is thought to influence systemic immune responses through mucosal immune mechanisms linking the upper respiratory tract mucosa to systemic inflammatory signalling [6]. Dysbiosis of the microbial community may promote chronic low-grade inflammation, complement system activation, and cytokine production, processes that are considered key components in the pathogenesis of AMD [7].

In addition, microorganisms and their metabolites can modulate host metabolic pathways, including tryptophan metabolism, which is closely linked to immune regulation, oxidative stress, and angiogenesis [8]. Such metabolic and immunological signals may affect distant tissues, including the retina, and thereby potentially contribute to retinal pigment epithelium damage, complement activation, and the development of choroidal neovascularisation [7].

The multifactorial character of AMD involves a complex interaction between genetic and environmental factors. The cumulative effects of the genome and its interactions with environmental exposures are captured by metabolomics, the study of metabolites (<1–1.5 kDa) [9]. Metabolites are small biological molecules involved in energy conversion and biosynthesis [10]. Beyond providing substrates, they provide vital signals that influence metabolic pathways or modulate regulatory proteins, including immune activation, cytokine release, and cell survival [11].

The kynurenine pathway represents the principal route of tryptophan degradation and generates biologically active metabolites involved in immune regulation and oxidative stress [12]. Conversely, tryptophan can be metabolised via the serotonin biosynthesis pathway, where L-5-hydroxytryptophan (5-HTP) serves as an important intermediate synthesised via the enzyme tryptophan hydroxylase (TPH). Through decarboxylation, 5-HTP transforms into serotonin (5-HT), which eventually becomes melatonin [13,14,15].

Because the conversion of tryptophan to 5-HTP is the rate-limiting step, it is foundational for maintaining neurological and metabolic health [16,17]. During aging or chronic inflammation, the shift toward the kynurenine pathway decreases 5-HTP and melatonin, reducing the eye’s natural defenses while increasing neurotoxic metabolites associated with retinal cell death [18,19]. Evaluating the link between these pathways within a shared metabolic “hub” allows for a new understanding of how nasopharyngeal signals influence retinal health through systemic circulation.

## 2. Methods

### 2.1. Establishment of the Study Cohorts

The study was carried out in accordance with the principles of the Declaration of Helsinki. Ethical approval was granted by the Kaunas Regional Biomedical Research Ethics Committee of the Lithuanian University of Health Sciences (approval No. BE-2-/48).

All participants underwent a thorough ophthalmological examination, during which information on general health and comorbidities was systematically collected. Written informed consent was obtained from each participant before inclusion in the study. Participants were then divided into two groups: those diagnosed with age-related macular degeneration (AMD) and ophthalmologically healthy control subjects. The diagnosis of AMD was confirmed by an ophthalmologist.

Patients were included in the AMD group if they were between 50 and 99 years old, had a confirmed diagnosis of either early or late AMD, and provided written informed consent to participate in the study. The diagnosis of exudative AMD was primarily based on optical coherence tomography (OCT) findings, with fluorescein angiography performed when necessary to support the diagnosis. AMD classification adhered to the criteria proposed by the Age-Related Eye Disease Study Research Group (2001) [20]. Individuals were excluded from the AMD group if they had ocular conditions unrelated to AMD that could affect macular morphology or interfere with retinal imaging interpretation. These included epiretinal membrane, macular hole, pathological myopia (including myopic maculopathy), central serous chorioretinopathy, vitelliform dystrophy, as well as high refractive error, corneal opacity, lens opacities (including nuclear, cortical, or posterior subcapsular cataracts, except for mild opacities), keratitis, acute or chronic uveitis, glaucoma, or optic nerve disorders.

To minimize potential confounding effects related to prior ocular interventions, patients were also excluded if they had undergone intraocular surgery, including cataract surgery, within the previous 3 months, or if they had a history of vitreoretinal surgery, including vitrectomy. In addition, patients who had received intravitreal anti-vascular endothelial growth factor (anti-VEGF) therapy within the previous 3 months were excluded to reduce the potential influence of recent treatment on retinal morphology and systemic metabolic profiles.

Participants were also excluded if they had systemic conditions, including diabetes mellitus, malignant tumours, systemic connective tissue diseases, chronic infectious diseases, or a history of organ or tissue transplantation. Additionally, patients whose colour fundus photographs could not be assessed due to media opacity or poor image quality were excluded from the study.

Control subjects were ophthalmologically healthy individuals aged from 50 to 99 years with no history of chronic infectious or non-infectious diseases who provided written informed consent to participate. Individuals were excluded from the control group if any ocular pathology was detected, including epiretinal membrane, macular hole, pathological myopia, central serous chorioretinopathy, or other macular disorders, or if OCT images could not be properly evaluated due to optical media opacity.

Control participants were also excluded if they had undergone intraocular surgery within the previous 3 months or had a history of vitreoretinal surgery. Furthermore, individuals with systemic diseases such as malignant tumours, connective tissue disorders, chronic infectious diseases, diabetes mellitus, or a history of organ or tissue transplantation were excluded. Participants currently using antiepileptic or sedative medications were also excluded.

### 2.2. Metabolite Profiling

Blood was mixed with an ice-cold methanol/water solution (final methanol concentration 50%) to extract metabolites, denature proteins, and slow down chemical reactions. Known quantities of internal standards (HEPES and PIPES) were added to the mixture. Metabolites were then separated by filtration through a 10 kDa cutoff filter to remove high-molecular-weight compounds. The prepared serum samples collected in tubes without anticogulant, were subsequently sent to Japan on dry ice under frozen conditions to preserve metabolite stability. Similarly, for blood-derived metabolites, serum samples were subjected to protein precipitation using an ice-cold methanol–water solution at a defined sample-to-solvent ratio (e.g., 1:4), followed by vortex mixing and centrifugation (e.g., ~14,000× *g* for 10–15 min at 4 °C) to remove precipitated proteins. The resulting supernatant was then processed using a 10 kDa molecular weight cut-off filter to eliminate residual high-molecular-weight components prior to LC–MS analysis.

The extracted serum samples were evaporated under a gentle stream of nitrogen to concentrate metabolites and facilitate solvent exchange prior to analysis. Chromatographic separation was performed using a ZIC-HILIC high-performance liquid chromatography (HPLC) column (150 × 2.1 mm, 3.5 µm) optimized for polar compounds.

Mass spectrometric analysis was performed using a high-resolution Orbitrap mass spectrometer equipped with an electrospray ionization (ESI) source operating in both positive and negative ion modes. Full-scan data were acquired at a resolution of 70,000 (at *m*/*z* 200) over a mass range of *m*/*z* 70–1000. The ion source voltage was set to 3.5 kV in positive mode and −2.5 kV in negative mode. The instrument was externally calibrated prior to each analytical batch using a standard calibration solution to ensure high mass accuracy throughout the analysis. A pooled quality control sample, generated by combining aliquots from the study samples, was analyzed at regular intervals throughout the analytical sequence to monitor instrument stability and signal drift.

The data obtained from HPLC–mass spectrometry were processed and analyzed using MZmine 2 software [21]. Metabolite profiles were measured in blood. Subsequent data analysis and visualization were performed using R scripts [21]. However, further analysis focused specifically on two intermediates of the tryptophan metabolic pathway—kynurenine and 5-hydroxytryptophan (5-HTP). These metabolites were selected due to their well-established roles in the regulation of immune responses, inflammatory processes, and host–microbiome interactions, which are considered important components in the pathogenesis of AMD. Considering the aim of this study to explore the interplay between microbiota and host metabolic pathways, the selection of these two metabolites enabled a focused evaluation of a biologically meaningful metabolic axis—the balance between the kynurenine and serotonin branches of tryptophan metabolism. In addition, these two metabolites were selected to represent the key branching point between the serotonin and kynurenine pathways of tryptophan metabolism [12,13,14,15], allowing assessment of pathway directionality rather than downstream pathway complexity. Although additional kynurenine pathway intermediates were detected in the metabolomic dataset, they were not included in the present analysis in order to reduce multiple testing burden and maintain a focused hypothesis-driven evaluation of microbiota-associated metabolic shifts. Other detected metabolites were not included in the subsequent analysis to limit the number of multiple comparisons, reduce the risk of statistical error, and maintain interpretative clarity, while focusing on metabolites whose pathogenetic mechanisms are most strongly linked to AMD, particularly through pathways involved in inflammation, immune regulation, and microbiome–host interactions.

In our previous study, we characterized the nasopharyngeal microbiota in patients with AMD. Here, we extend these findings by integrating microbiome profiling with systemic metabolic analysis, focusing on two key metabolites of the tryptophan pathway—kynurenine and 5-hydroxytryptophan (5-HTP). This approach aimed to determine whether nasopharyngeal microbial communities are associated with alterations in tryptophan metabolism, a pathway closely linked to immune regulation, inflammation, and neurovascular processes implicated in AMD pathogenesis.

### 2.3. Statistical Analysis

Statistical analyses were performed using R software (version 4.1.2; R Foundation for Statistical Computing, Vienna, Austria). Continuous variables were assessed for distribution and summarized as mean ± standard deviation (SD) or median with interquartile range (IQR) as appropriate, while categorical variables were expressed as counts and percentages.

Comparisons between patients with exudative AMD and control subjects were conducted using Student’s *t*-test for continuous variables and the chi-square test for categorical variables.

Metabolite concentrations were log_2_-transformed before analysis to enhance distributional properties and lessen skewness. Pearson correlation analysis was conducted to assess the relationship between 5-HTP and kynurenine levels.

To assess the relationship between metabolites and AMD status, logistic regression models were developed. Odds ratios (OR) and their 95% confidence intervals (CI) were computed. Multivariable models were adjusted for age and gender to control for possible confounding factors.

The diagnostic performance of metabolites was evaluated using receiver operating characteristic (ROC) curve analysis, and the area under the curve (AUC) was determined to assess the discrimination between AMD patients and controls. ROC curves were plotted for individual metabolites, their combined model, and the kynurenine/5-HTP ratio, as well as for models adjusted for demographic variables.

Optimal classification thresholds were identified using the Youden index, and diagnostic accuracy was expressed through sensitivity and specificity.

All statistical tests were two-sided, and *p*-values < 0.05 were considered statistically significant.

Data visualization included boxplots for group comparisons, scatter plots with regression lines for correlation analyses, and ROC curves for evaluation of diagnostic performance.

## 3. Results

### 3.1. Baseline Characteristics of Study Participants

The study included 232 participants, comprising 147 control subjects and 85 patients with exudative age-related macular degeneration (AMD). Baseline demographic characteristics and plasma metabolite concentrations are summarized in Table 1.

A substantial age difference was observed between the two groups. Patients with AMD were significantly older than control subjects (77.07 ± 7.94 vs. 50.36 ± 20.56 years, *p* < 0.001). The gender distribution was comparable between groups, with females representing 69.4% of controls and 62.4% of AMD patients, and males 30.6% and 37.6%, respectively (*p* = 0.27), indicating no significant difference in gender composition.

### 3.2. Differences in Tryptophan Metabolites Between AMD Patients and Controls

Plasma levels of two tryptophan metabolism intermediates, 5-hydroxytryptophan (5-HTP) and kynurenine, were compared between patients with exudative AMD and control subjects. Log_2_-transformed metabolite levels were used for statistical analysis.

**Figure 1:**
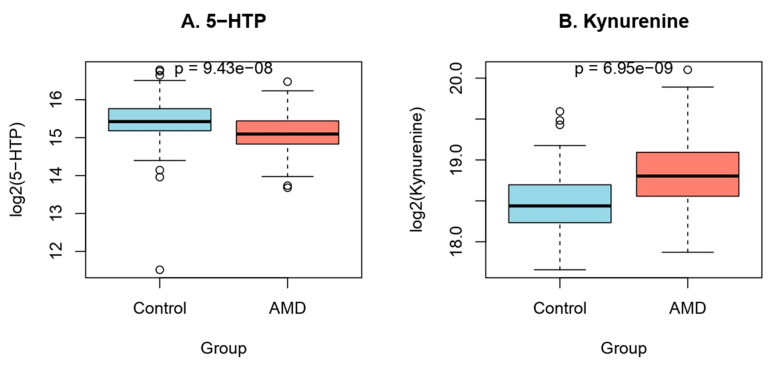
Altered tryptophan metabolism in exudative AMD. Boxplots showing log_2_-transformed plasma concentrations of (**A**) 5-hydroxytryptophan (5-HTP) and (**B**) kynurenine in control subjects and patients with exudative age-related macular degeneration (AMD). Whiskers show the range without outliers, while boxes show the interquartile range (IQR), with the median shown by the horizontal line. The Wilcoxon rank-sum test was used to determine the statistical significance between groups.

AMD patients exhibited significantly lower levels of 5-HTP and significantly higher levels of kynurenine compared with controls (Wilcoxon rank-sum test, *p* < 0.001 for both comparisons), indicating alterations in tryptophan metabolism associated with the disease (Figure 1).

### 3.3. Logistic Regression Analysis of Individual Metabolites

To evaluate the independent contribution of each metabolite to AMD status, logistic regression analysis was performed using log_2_-transformed metabolite levels as predictors. Both metabolites were significantly associated with AMD. Higher kynurenine levels were associated with an increased probability of AMD (β = 3.05, *p* = 1.98 × 10^−10^), whereas higher 5-HTP levels were associated with a reduced probability of AMD (β = −2.20, *p* = 7.27 × 10^−8^). These findings indicate that both metabolites contribute independently to disease classification.

### 3.4. Diagnostic Performance of Metabolite Biomarkers

**Figure 2:**
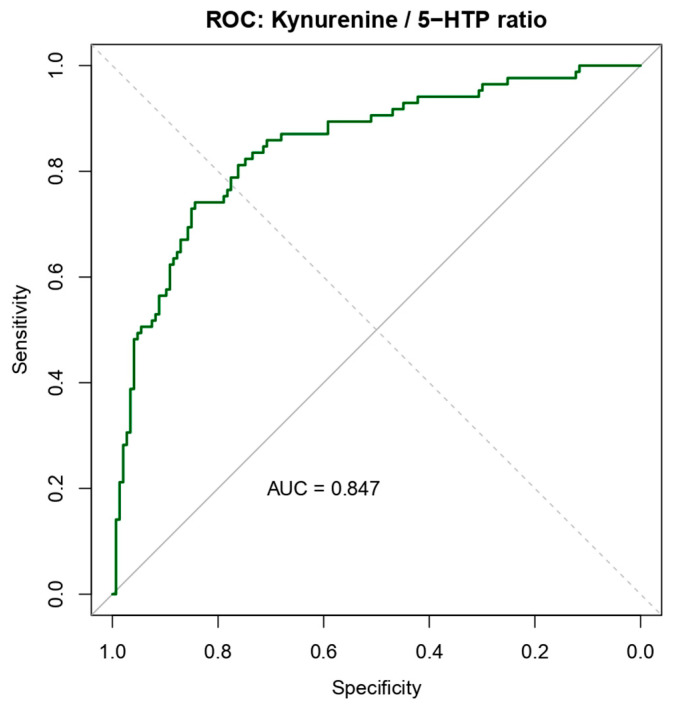
Diagnostic performance of tryptophan metabolites for AMD detection. Receiver operating characteristic (ROC) curves illustrate the ability of plasma tryptophan metabolism markers to distinguish patients with exudative age-related macular degeneration (AMD) from control subjects. The combined metabolite model demonstrates improved diagnostic performance compared with individual metabolites.

Receiver operating characteristic (ROC) curve analysis was performed to evaluate the ability of tryptophan metabolism markers to discriminate patients with exudative AMD from control subjects (Table 2, Figure 2).

When analyzed individually, both metabolites demonstrated moderate diagnostic performance. Plasma 5-HTP yielded an area under the curve (AUC) of 0.71, while kynurenine showed a similar discriminatory ability with an AUC of 0.73.

Combining both metabolites in a logistic regression model improved diagnostic accuracy, resulting in an AUC of 0.84, indicating good discrimination between AMD patients and controls. A similar level of performance was observed for the kynurenine/5-HTP ratio, which achieved an AUC of 0.85.

Further improvement was observed after adjusting the ratio model for age and gender, which increased the AUC to 0.92, indicating excellent diagnostic performance. These findings suggest that alterations in the tryptophan metabolic pathway, particularly the balance between kynurenine and 5-HTP, provide strong discriminatory power for identifying individuals with exudative AMD.

### 3.5. Kynurenine/5-HTP Ratio as an Indicator of Pathway Activity

**Figure 3:**
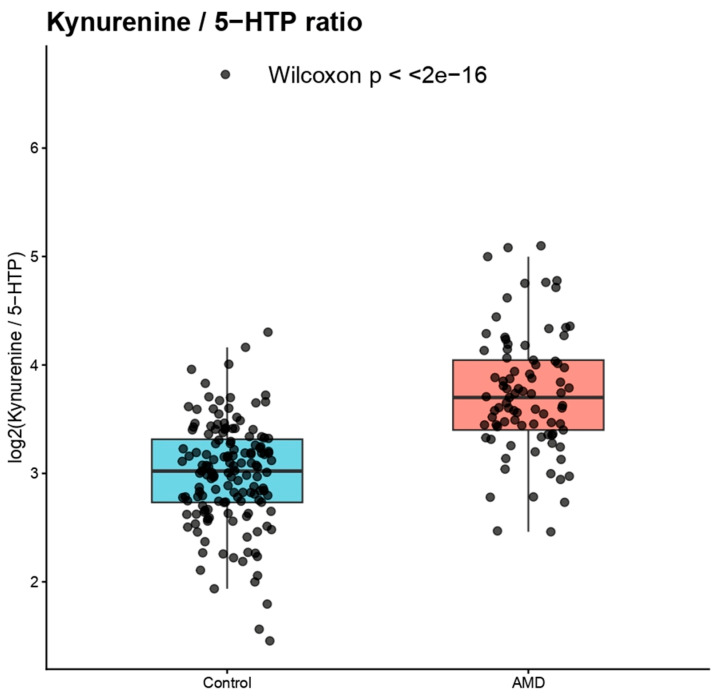
Kynurenine/5-hydroxytryptophan ratio in AMD and control groups. Boxplot showing the distribution of the kynurenine/5-HTP ratio in AMD patients and control subjects. The ratio was significantly elevated in AMD, indicating increased activation of the kynurenine pathway.

The kynurenine/5-HTP ratio was computed as a measure of pathway activity as both metabolites are part of the tryptophan metabolic pathway. When compared to controls, the ratio was significantly higher in AMD patients (Wilcoxon test, *p* < 2.2 × 10^−16^), suggesting a marked change in tryptophan metabolism toward the kynurenine pathway (Figure 3).

ROC analysis demonstrated strong discriminatory performance for this ratio (AUC = 0.847, 95% CI 0.794–0.900). The optimal threshold value identified using the Youden index was 3.43, corresponding to 74.1% sensitivity and 84.4% specificity.

### 3.6. Metabolic Signature of AMD

**Figure 4:**
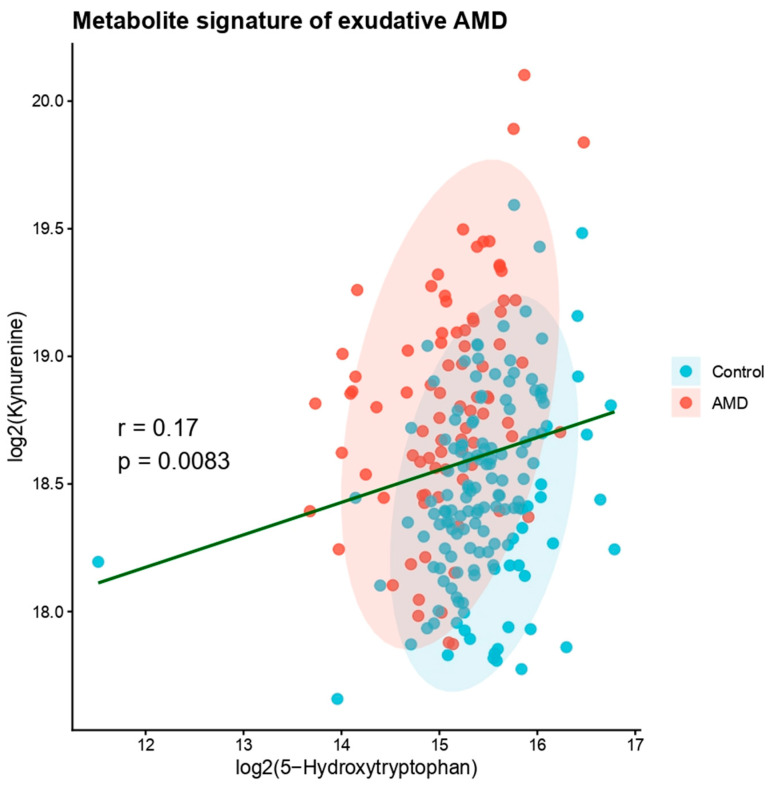
Metabolite signature of exudative AMD. Scatter plot showing log_2_-transformed plasma 5-hydroxytryptophan and kynurenine levels in control subjects and patients with exudative AMD. Colored ellipses indicate the 95% distribution of each group. The fitted regression line is shown in green, and the inset indicates the Pearson correlation between metabolites.

5-HTP and kynurenine levels were generated. AMD patients displayed a distinct metabolic profile characterised by reduced 5-HTP and increased kynurenine levels, forming a characteristic metabolic signature associated with the disease (Figure 4).

### 3.7. Association of the Kynurenine/5-HTP Ratio with AMD After Adjustment for Age and Gender

To determine whether the observed metabolic alterations were independent of demographic factors, age- and gender-adjusted logistic regression analysis was carried out. The kynurenine/5-HTP ratio remained significantly associated with AMD (β = 1.84, *p* = 4.15 × 10^−6^). Each unit increase in the ratio was associated with a 6.30-fold increase in the odds of AMD (95% CI 3.08–14.81). Age was also significantly linked to AMD (OR = 1.12, *p* = 7.11 × 10^−9^), whereas gender showed no significant effect (Table 3).

ROC analysis of the adjusted model demonstrated excellent discrimination between AMD patients and controls, with an AUC of 0.923.

## 4. Discussion

This study demonstrates significant alterations in tryptophan metabolism in patients with exudative AMD. Specifically, exudative AMD patients exhibited reduced plasma levels of 5-hydroxytryptophan (5-HTP) and increased levels of kynurenine, indicating a shift in tryptophan metabolism toward the kynurenine pathway. Importantly, the kynurenine/5-HTP ratio was markedly elevated in exudative AMD patients, suggesting enhanced activation of this metabolic pathway. Logistic regression analysis confirmed that the kynurenine/5-HTP ratio was strongly associated with AMD independently of age and gender, with a more than six-fold increase in the odds of AMD per unit increase in the ratio. Furthermore, ROC analysis demonstrated excellent discriminatory performance for the adjusted model, with an AUC exceeding 0.92.

**Figure 5:**
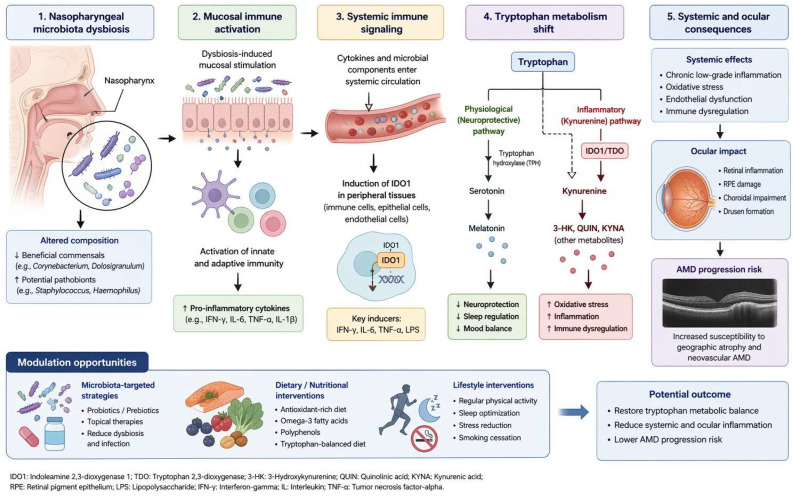
Conceptual model linking nasopharyngeal microbiota and tryptophan metabolism. Alterations in nasopharyngeal microbiota may promote mucosal immune activation and cytokine release, leading to induction of IDO1 and a metabolic shift of tryptophan toward the kynurenine pathway. This shift may contribute to systemic inflammation and oxidative stress, potentially influencing AMD pathogenesis.

Tryptophan metabolism plays a central role in immune regulation and inflammatory signaling [22]. Under physiological conditions, tryptophan is metabolized through multiple pathways, including serotonin synthesis and degradation via the kynurenine pathway. Activation of the kynurenine pathway is mediated by indoleamine 2,3-dioxygenase (IDO1) and tryptophan 2,3-dioxygenase (TDO), which are induced by inflammatory stimuli [23]. Emerging evidence suggests that mucosal microbiota alterations, including those in the nasopharyngeal environment, may contribute to this metabolic shift by promoting local and systemic immune activation, leading to increased production of pro-inflammatory cytokines such as interferon-γ and interleukin-6. These cytokines are known to upregulate IDO1 expression, thereby driving tryptophan metabolism toward the kynurenine pathway. Elevated kynurenine levels, therefore, often reflect chronic immune activation and inflammatory processes, both of which are central features of AMD pathophysiology. Previous studies have demonstrated that inflammatory mediators, oxidative stress, and complement activation contribute to retinal degeneration in AMD [7]. Our findings are consistent with these mechanisms and suggest that altered tryptophan metabolism may represent an additional metabolic signature associated with disease progression. This proposed mechanism is summarized in Figure 5.

**Figure 6:**
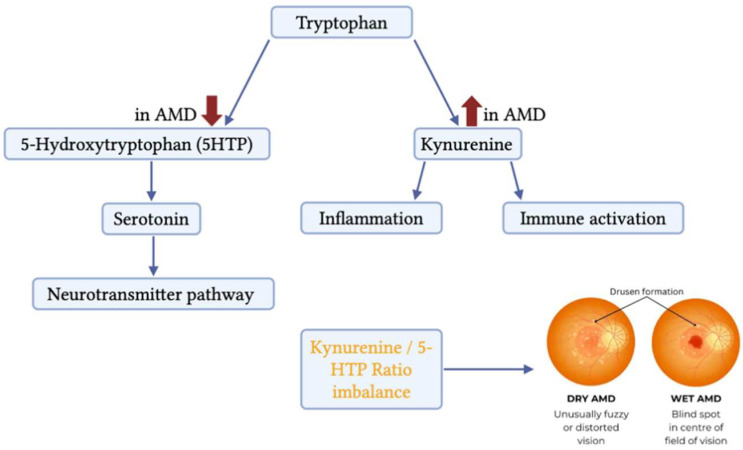
Proposed metabolic shift in tryptophan metabolism associated with exudative AMD. Tryptophan can be metabolized through the serotonin pathway, producing 5-hydroxytryptophan (5-HTP), or via the kynurenine pathway, which is connected with inflammatory and immune activation processes. In patients with exudative AMD, plasma 5-HTP levels were reduced, while kynurenine levels were elevated, resulting in an increased kynurenine/5-HTP ratio. This shift toward the kynurenine pathway may reflect inflammatory activation and potential interactions between host metabolism and microbial communities contributing to AMD pathogenesis.

The observed decrease in 5-HTP levels may also have biological relevance. 5-HTP is an intermediate in serotonin synthesis and plays an important role in neuronal signaling and oxidative stress regulation. Reduced levels of this metabolite could reflect impaired serotonin pathway activity or increased diversion of tryptophan toward the kynurenine pathway under inflammatory conditions. Such metabolic reprogramming has been described in several chronic inflammatory and neurodegenerative diseases, supporting the hypothesis that AMD shares metabolic features with systemic inflammatory disorders [16]. Although the present study focused specifically on kynurenine and 5-hydroxytryptophan as representative intermediates of the two principal branches of tryptophan metabolism, additional downstream kynurenine pathway metabolites such as kynurenic acid, 3-hydroxykynurenine, and quinolinic acid may provide further insight into the balance between neuroprotective and neurotoxic pathway activity [5]. Previous studies have suggested that alterations in these downstream metabolites reflect shifts toward the neurotoxic arm of the kynurenine pathway in AMD [5]. Future studies incorporating a broader panel of kynurenine pathway intermediates may therefore help us to better localize where along the pathway the metabolic shift is most pronounced and further refine the biological interpretation of microbiota-associated metabolic alterations. Our findings are further supported by recent evidence highlighting the role of nutritional interventions in modulating inflammatory and metabolic pathways relevant to AMD. In particular, a recent study by Josifova et al. demonstrated that targeted dietary strategies may influence disease progression through regulation of inflammation and metabolic homeostasis, reinforcing the concept that modulation of tryptophan metabolism and microbiota-related pathways may represent a promising approach for AMD risk reduction and management [24]. Moreover, our findings may also be linked to microbial influences on host metabolism. In our previous microbiome study, we identified increased abundance of *Corynebacterium* species in AMD patients, particularly among individuals with poor response to anti-VEGF therapy. Members of the *Corynebacterium* genus are known to influence host immune responses [25] and metabolic processes, including amino acid metabolism [26]. Microbial communities can modulate the balance between the serotonin and kynurenine pathways by influencing host immune signaling and inflammatory responses [27]. Although the metabolite and microbiome analyses were conducted in separate cohorts, the metabolic shift toward increased kynurenine levels and an elevated kynurenine/5-HTP ratio observed in this study may reflect host–microbiome interactions affecting tryptophan metabolism [28,29,30]. Activation of the kynurenine pathway has previously been linked to chronic inflammation, immune activation, and neurodegeneration—processes that are increasingly recognised as key contributors to retinal degeneration in AMD [5,31]. A schematic representation of the proposed relationship between altered tryptophan metabolism and AMD pathophysiology is presented in Figure 6.

From a clinical perspective, the kynurenine/5-HTP ratio demonstrated strong diagnostic potential. The ratio alone showed good discriminatory ability (AUC ≈ 0.85), while adjustment for age and gender improved the model performance further (AUC ≈ 0.92). These findings suggest that tryptophan metabolism markers may serve as potential blood-based biomarkers for AMD. Blood biomarkers are of particular interest in AMD research because current diagnosis relies primarily on imaging-based retinal assessments. Metabolic biomarkers could therefore complement clinical evaluation and potentially assist in disease risk stratification or monitoring [5].

Research on the nasopharyngeal or upper respiratory tract microbiota in AMD remains poorly characterized. Ho et al., in a large cohort study including 245 AMD patients and 386 control subjects, demonstrated that the human pharyngeal microbiota may be associated with AMD and identified candidate bacterial genera such as *Prevotella* and *Gemella* that may be linked to disease development and progression [32]. Similarly, Rullo et al. [33], in a case–control study comparing the nasal microbiota of 13 patients with newly diagnosed neovascular AMD and 5 control individuals, reported significant differences in microbial composition between groups, including an increased abundance of taxa belonging to the phylum *Actinobacteriota* in the patient group.

The traditional understanding of AMD is undergoing a paradigm shift, moving from a localized ocular perspective to a systemic model defined by the nasopharyngeal–retina axis. While the condition is the primary driver of irreversible vision loss in the elderly, affecting over 13% of those aged 85 and older, its origins appear to be deeply rooted in the metabolic and microbial ecology of the upper respiratory tract. Recent large-scale shotgun sequencing of the pharyngeal microbiome has revealed that while a “core” community of *Streptococcus* and *Prevotella* maintains overall stability, significant dysbiosis emerges as the disease progresses from early to late stages [34,35,36,37,38].

This dysbiosis is characterized by a “metabolic tug-of-war” within a shared biochemical hub. In a healthy state, the nasopharyngeal environment supports the conversion of tryptophan into L-5-hydroxytryptophan (5-HTP), the critical precursor to serotonin and melatonin. Melatonin acts as a vital antioxidant shield for retinal pigment epithelium (RPE) cells. However, in AMD cases, the microbiome undergoes a shift—marked by a significant increase in *Gemella* and a depletion of *Prevotella*—which correlates with a systemic diversion of tryptophan toward the kynurenine pathway. This shift reduces protective melatonin levels while increasing pro-inflammatory metabolites such as 3-hydroxykynurenine, which promote oxidative stress and pathological angiogenesis seen in “wet” AMD [34,39,40]. The complexity of this axis is further deepened by host genetics and community interactions. Research indicates that certain driver genera, such as *Gemella*, do not act in isolation but form symbiotic clusters with other bacteria, such as *Haemophilus* and *Aggregatibacter*, potentially amplifying their pathogenic impact. Furthermore, host genetic variants, such as those in the *Complement Factor H* (*CFH*) gene, may alter how the mucosal immune system interacts with these bacteria, potentially allowing certain microbes to evade immune detection and perpetuate chronic systemic inflammation [41,42,43,44]. Although these findings suggest that the composition of the nasopharyngeal microbiota may be related to biological processes involved in AMD, the available evidence remains limited.

In the study by Dundar et al. [5], serum metabolites of the tryptophan–kynurenine pathway (TRP, KYN, KYNA, 3HK, 3HAA, and QA) were assessed in patients with AMD and healthy controls. The authors reported significantly elevated levels of 3-hydroxykynurenine in AMD patients, whereas kynurenine, tryptophan, quinolinic acid, and the KYNA/3HK ratio were higher in the control group. These findings indicate a shift of the kynurenine pathway towards the neurotoxic branch, reflected by increased 3HK and a lowered KYNA/3HK ratio. Such changes point to an imbalance between neuroprotective and neurotoxic metabolites and support the hypothesis that dysregulation of the kynurenine pathway may contribute to the development of AMD. These findings further support the relevance of downstream kynurenine pathway metabolites for distinguishing between neuroprotective and neurotoxic pathway activation states and complement the interpretation of upstream metabolic shifts observed in the present study [5]. Tryptophan metabolism may vary between genders, as demonstrated by Pais et al. [45], who showed that concentrations of several kynurenine pathway metabolites differ between males and females. These differences are likely driven by hormonal regulation, especially by estrogen and other gender hormones, affecting key enzymes involved in tryptophan breakdown, such as indoleamine-2,3-dioxygenase and tryptophan-2,3-dioxygenase. This metabolic variability may also be significant in the context of AMD, as epidemiological studies [46] suggest a slightly higher risk of the disease in women compared to men (OR = 1.2; 95% CrI, 1.0–1.5). Although gender-related differences in AMD prevalence are not entirely consistent across populations, several studies indicate that women may bear a greater disease burden, particularly in older age groups. These observations imply that biological gender–related metabolic differences, including variations in tryptophan metabolism and immune regulation, could influence disease susceptibility and progression, as well as the variability of metabolic biomarker profiles observed in AMD patients [45].

L-5-hydroxytryptophan (5-HTP) is synthesised from tryptophan through the action of tryptophan hydroxylase (TPH). It is subsequently decarboxylated to form serotonin (5-hydroxytryptamine, 5-HT), a monoamine neurotransmitter that plays a key role in regulating mood, cognition, reward, learning, memory, sleep, and many other physiological functions [13]. The level of endogenous 5-HTP available for serotonin production is influenced by both tryptophan availability and the activity of several enzymes, particularly TPH, indoleamine 2,3-dioxygenase, and tryptophan 2,3-dioxygenase (TDO) [47]. In individuals with depression, the concentrations of tryptophan, serotonin, kynurenine, and their related metabolites are still not fully understood. Serotonin is known to be involved in the pathophysiology of depression, and research suggests that the transport of 5-HTP across the blood–brain barrier may be impaired in major depressive disorder [48], including cases observed in childhood depression [49]. Early investigations highlighted the potential antidepressant effects of both tryptophan and 5-HTP [50], and their use has generally been associated with relatively few side effects in depressed patients [51]. However, 5-HTP administration can lead to dose-dependent gastrointestinal disturbances. When combined with a peripheral decarboxylase inhibitor, it may also produce psychological side effects such as acute anxiety [52]. Higher doses (above 100 mg) have been associated with nausea and vomiting [53]. Additionally, 5-HTP has been identified as a useful option for migraine prevention [54,55]. In individuals prone to headaches, it may help modulate central nervous system (CNS) dysfunctions involved in migraine mechanisms. One study reported a significant reduction in headache days after 2 weeks of 5-HTP treatment [56].

Ataxia is a clinical sign indicating impaired function in regions of the nervous system responsible for coordinating movement, particularly the cerebellum. Some aspects of cerebellar ataxia have been shown to partially improve after prolonged administration of 5-HTP [57], with notable reductions in kinetic scores that suggest improved coordination, although the benefits may not be complete in all cases [58]. Reduced levels of 5-HTP have been observed in women with fibromyalgia (FM) compared to healthy controls, implying that disturbances in catecholamine and indolamine pathways may contribute to the disorder’s pathophysiology [59,60]. Treatment with 5-HTP has been linked to significant improvements in multiple clinical outcomes among 50 patients with primary FM syndrome, with only mild and temporary side effects reported [61]. In dementia of the Alzheimer’s type (DAT), concentrations of 5-HTP in cerebrospinal fluid (CSF) are lower than those seen in control subjects. This suggests involvement of the serotonergic system in DAT and indicates potential diagnostic relevance [62]. 5-HTP has also been used for many years as a therapeutic approach in Parkinson’s disease (PD) [63,64]. In patients with Parkinsonism, co-administration of L-dopa with 5-HTP and decarboxylase inhibitors does not appear to affect the gastrointestinal absorption of 5-HTP [65]. Moreover, a single-centre, randomised, double-blind, placebo-controlled, crossover study found that patients taking 50 mg of 5-HTP daily for four weeks experienced a significant reduction in depressive symptoms compared to placebo, providing preliminary evidence for its potential in managing depression in PD [66].

When evaluating the results of this study, several limitations should be taken into account. First, the cross-sectional design limits the ability to infer causal relationships between alterations in metabolite levels and disease progression. The present study demonstrates that systemic changes in tryptophan metabolism are associated with AMD and emphasizes the potential importance of the kynurenine pathway as a metabolic indicator of the disease. Secondly, although major systemic diseases known to significantly affect metabolic profiles (such as diabetes mellitus, malignant tumours, chronic infectious diseases, connective tissue disorders, and post-transplantation status) were excluded according to the study protocol, several additional factors that may influence metabolite levels, including dietary habits, use of nutritional supplements (including AREDS formulations), smoking status, medication use, and previous anti-VEGF treatment, were not assessed in the present study and therefore could not be included as covariates in the statistical analysis. These factors should be considered when interpreting the metabolomic findings. A further limitation of the present study is that the diagnostic performance of the kynurenine/5-hydroxytryptophan (5-HTP) ratio was evaluated within a single cohort, and the proposed threshold has not been validated in an independent population. As a result, the observed discriminatory ability may be subject to overestimation due to cohort-specific characteristics. Although the kynurenine/5-HTP ratio demonstrated promising potential as a biomarker of altered tryptophan metabolism in AMD, its clinical applicability remains to be established. Future studies should aim to validate these findings in larger, independent cohorts to assess the robustness and generalizability of the proposed threshold. In addition, internal validation approaches, such as cross-validation or bootstrapping, may provide further insight into the stability of the observed associations and help mitigate the risk of overfitting. Overall, further investigations are needed to clarify whether these metabolic alterations primarily reflect systemic inflammatory processes, retinal metabolic disturbances, or interactions between host metabolism and microbial communities.

## 5. Conclusions

Exudative AMD is associated with a distinct metabolic shift toward the kynurenine pathway, characterized by decreased 5-HTP levels, increased kynurenine, and an elevated kynurenine/5-HTP ratio. This ratio showed a strong and independent association with AMD and demonstrated excellent discriminatory performance. These findings suggest that the tryptophan–kynurenine metabolic axis may play an important role in AMD pathogenesis and highlight its potential as a biomarker and a target for future investigation.

## Figures and Tables

**Table 1:** Clinical and metabolite characteristics of study participants.

Variable	Control (*n* = 147)	AMD (*n* = 85)	*p*-Value
Age, years (mean ± SD)	50.36 ± 20.56	77.07 ± 7.94	<0.001
Female, *n* (%)	102 (69.4)	53 (62.4)	0.27
Male, *n* (%)	45 (30.6)	32 (37.6)	

Mean ± standard deviation (SD) is used to present age. Counts and percentages are used to present categorical variables. Student’s *t*-test for age and the χ^2^ test for categorical data were used to compare groups.

**Table 2:** Diagnostic performance of tryptophan pathway metabolites for AMD detection.

Model	AUC	Interpretation
5-Hydroxytryptophan (5-HTP)	0.71	Moderate discrimination
Kynurenine	0.73	Moderate discrimination
5-HTP + Kynurenine	0.84	Good discrimination
Kynurenine/5-HTP ratio	0.85	Good discrimination
Ratio + age + gender	0.92	Excellent discrimination

Diagnostic performance of plasma tryptophan metabolism markers for distinguishing patients with exudative age-related macular degeneration (AMD) from control subjects. Diagnostic accuracy was evaluated using receiver operating characteristic (ROC) curve analysis. AUC values indicate the ability of each biomarker or model to discriminate AMD cases from controls.

**Table 3:** Multivariable logistic regression analysis of factors linked to exudative AMD.

Variable	Odds Ratio (OR)	95% CI	*p*-Value
Kynurenine/5-HTP ratio	6.30	3.08–14.81	4.15 × 10^−6^
Age	1.12	1.08–1.17	7.11 × 10^−9^
Male	1.32	0.57–3.08	0.512

Multivariable logistic regression model evaluating the association between the kynurenine/5-hydroxytryptophan ratio and AMD after adjustment for age and gender. Odds ratios (OR) with 95% confidence intervals (CI) are reported.

## Data Availability

The datasets used and/or analysed during the current study are available from the corresponding author on reasonable request.
